# Proteomic analysis at the sites of clinical infection with invasive *Streptococcus pyogenes*

**DOI:** 10.1038/s41598-018-24216-2

**Published:** 2018-04-13

**Authors:** Robert J. Edwards, Marta Pyzio, Magdalena Gierula, Claire E. Turner, Vahitha B. Abdul-Salam, Shiranee Sriskandan

**Affiliations:** 1 Faculty of Medicine, Imperial College London, London, U.K.; 2 Present Address: Molecular Biology & Biotechnology, Firth Court, University of Sheffield, Sheffield, U.K.

## Abstract

Invasive *Streptococcus pyogenes* infections are rare, with often-unexplained severity. Prompt diagnosis is desirable, as deaths can occur rapidly following onset and there is an increased, but preventable, risk to contacts. Here, proteomic analyses of clinical samples from invasive human *S*. *pyogenes* infections were undertaken to determine if novel diagnostic targets could be detected, and to augment our understanding of disease pathogenesis. Fluid samples from 17 patients with confirmed invasive *S*. *pyogenes* infection (empyema, septic arthritis, necrotising fasciitis) were analysed by proteomics for streptococcal and human proteins; 16/17 samples had detectable *S*. *pyogenes* DNA. Nineteen unique *S*. *pyogenes* proteins were identified in just 6/17 samples, and 15 of these were found in a single pleural fluid sample including streptococcal inhibitor of complement, trigger factor, and phosphoglycerate kinase. In contrast, 469 human proteins were detected in patient fluids, 177 (38%) of which could be identified as neutrophil proteins, including alpha enolase and lactotransferrin which, together, were found in all 17 samples. Our data suggest that streptococcal proteins are difficult to detect in infected fluid samples. A vast array of human proteins associated with leukocyte activity are, however, present in samples that deserve further evaluation as potential biomarkers of infection.

## Introduction

The important human pathogen *Streptococcus pyogenes* (group A *Streptococcus*, GAS) causes a spectrum of disease, from non-invasive throat infections to invasive necrotising fasciitis, pneumonia and septic shock. As most deaths from invasive GAS (iGAS) occur within 1–2 days of sample collection^1^, rapid diagnosis is required to enable prompt management and source control. Furthermore, although iGAS is rare, there is a 2000-fold increased risk of secondary cases of iGAS in household contacts, who may be protected if provided with timely advice^2^. Although iGAS can be diagnosed from blood cultures in around 80% of cases^3^, culture can take 24 h, and delays in diagnosis occur because samples from the site of infection may require specialist procedures including surgically- or radiologically-guided aspiration; patients may therefore be pre-exposed to systemic antimicrobials that will inhibit culture of microorganisms. Gram stains of samples can provide diagnoses, but lack sensitivity in septic arthritis compared with empyema^4,5^. There is therefore an urgent need for a sensitive rapid diagnostic test that can be undertaken on samples from patients with suspected iGAS after antimicrobials have started. Point-of-care rapid antigen tests for sore throat, that recognise the carbohydrate group antigen of *S*. *pyogenes*, can be adapted for use in iGAS infection^6,7^ but thus far no protein-based targets have been identified.

Several proteomic analyses of *S*. *pyogenes* have been reported, including both secreted and bacterial cell-associated proteomes following culture *in vitro*, in biofilm, and interactions with human plasma proteins^8–11^. The number of proteins detectable is often large and extends beyond those considered as classical virulence factors. Indeed, many metabolic so-called moonlighting proteins dominate the GAS secretome and could represent potentially important targets for immunodiagnostics. Similar to other bacteria, *S. pyogenes* may alter its proteome transiently in response to environment and growth phase; the precise growth phase of *S*. *pyogenes* during clinical invasive infection is uncertain. Furthermore, clinical infections are caused by a range of *emm* (M) genotypes that differ in regulatory networks and therefore may result in distinctive proteomes during infection. Indeed, this is apparent during broth culture^8^. Building on this knowledge, we set out to investigate whether *S*. *pyogenes* proteins could be detected in iGAS clinical samples submitted for routine culture to the diagnostic laboratory of a large teaching hospital, with the primary aim of identifying potentially novel diagnostic targets; and with a secondary aim of examining the host proteomic response to iGAS infection.

## Results

### Clinical samples

Seventeen samples from patients with iGAS were obtained; of these, 7 were from patients with necrotising fasciitis, 7 from patients with suspected septic arthritis (2 were from the same episode, separated by 24 h, in a single patient), and 3 from patients with empyema. All empyema cases were caused by *emm*1 *S*. *pyogenes* in children. Septic arthritis and necrotising fasciitis cases were associated with a wider range of leading *emm* types including *emm*1, *emm*4, *emm*28 and *emm*89 (Table 1).

**Table 1:** Characteristics of the human exudate samples collected for this study.

Fluid no.	Type of fluid	Infection	culture +/−	Isolate source	Isolate	*emm* type	^¶^GAS DNA (copies/µl)
F1	Tissue exudate	Necrotising fasciitis	+	Tissue	H897^§^	*emm*4	1.96E + 04
F2	Tissue exudate	Necrotising fasciitis	+	Tissue	H894^§^	*emm*28	7.10E + 05
F3*	Knee aspirate	Septic arthritis	+	Aspirate	H893	*emm*4	1.45E + 07
F4*	Knee aspirate	Septic arthritis	−	Aspirate	H892	*emm*4	9.73E + 04
F5	Pleural fluid	Pneumonia/Empyema	+	Aspirate	H899	*emm*1	1.45E + 07
F6**	Knee aspirate	Reactive arthritis	−	Blood	H832	*emm*28	ND
F7	Pleural fluid	Empyema	+	Aspirate	H842	*emm*1	1.64E + 04
F8	Pleural fluid	Empyema	+	Aspirate	H843	*emm*1	5.37E + 08
F9	Hip aspirate	Septic arthritis	+	Blood	H885^§^	*emm*77	3.52E + 08
F10	Knee aspirate	Septic arthritis	+	Aspirate	H657	*emm*89	3.23E+06
F11	Tissue exudate	Necrotising fasciitis	+	Tissue	H700^§^	*emm*89	6.11E+04
F12	Tissue exudate	Necrotising fasciitis	+	Tissue	H751	*emm*1	2.05E+05
F13	Tissue exudate	Necrotising fasciitis	+	Tissue	H758^§^	*emm*1	1.66E+06
F14	Elbow aspirate	Septic arthritis	+	Aspirate	H618^§^	*emm*28	1.23E+06
F15	Knee aspirate	Septic arthritis	−	Blood	H621	*emm*89	1.71E+04
F16	Tissue exudate	Necrotising fasciitis	+	Tissue	H627	*emm*4	1.33E+07
F17	Tissue exudate	Necrotising fasciitis	+	Tissue	H629^§^	*emm*1	1.09E+06

^¶^GAS copy number minimum detection level 6.79E + 02. Any value below this was not detectable (ND).

^*^Same patient; fluids aspirated 1 day apart F3 is first sample (H893); F4 was second (H892).

^**^Arthritis thought to be reactive.

^§^Isolates where culture supernatants were subject to separate proteomic analysis.

In all cases, *S*. *pyogenes* was cultured from a sterile site. In 14/17 cases the isolate was cultured from the fluid sample submitted for testing. In the remainder *S*. *pyogenes* was cultured from blood and was not cultured from the sample. Antibiotic exposure was not known for any of the samples. Quantitative PCR was undertaken as a surrogate of bacterial density and confirmed the presence of *S*. *pyogenes* DNA in all samples bar one, F6, where arthritis associated with bacteremia was subsequently thought to have been reactive. The median *S*. *pyogenes* genome copy number in the infected clinical fluids was 1.16 × 10^6^ copies/μl (range 1.64 × 10^4^–5.37 × 10^8^ copies/μl).

### SDS-polyacrylamide gel electrophoresis

**Figure 1:**
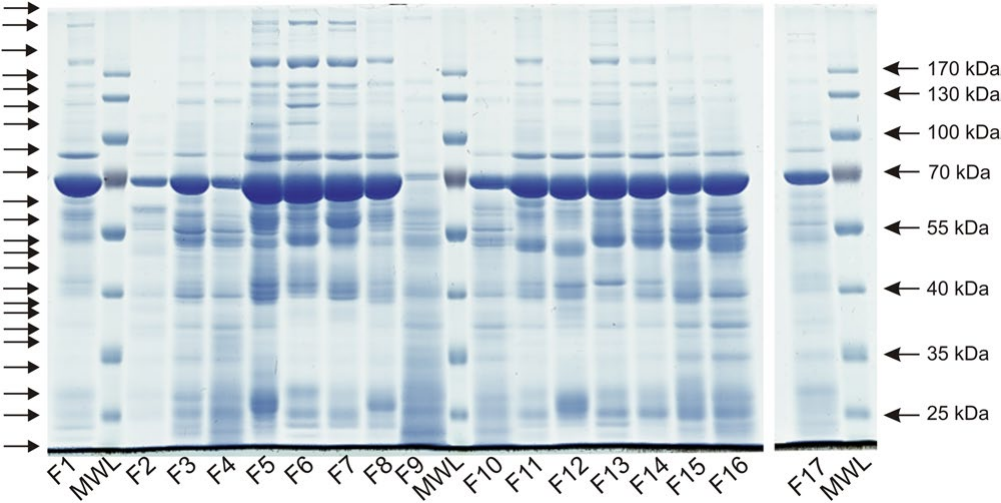
SDS-polyacrylamide gel separation of clinical fluid sample proteins. Clinical fluids F1–F17 were separated by electrophoresis along with a molecular weight ladder (MWL). Following staining with coomassie blue each lane of the gel was cut into 22 sections, as indicated by the arrows on the lefthand side, in preparation for proteomic analysis as described in the Methods section. For the purpose of clarity the image shown has been cropped to show the lanes corresponding to the clinical fluid samples. The uncropped image is shown as Supplementary Figure S1.

Separation of the clinical fluid sample by SDS-polyacrylamide gel electrophoresis showed that most of the samples had a similar profile of proteins albeit with much variation in intensities (Fig. 1). An intense band at just below 70 kDa, which would be consistent with albumin was present in all samples, except for F9 where it was difficult to discern and appeared to be reduced in F2, F4 and F10. All four of these samples also appeared to lack many of the higher mass proteins evident in the other fluid samples. In addition, F2 appeared to lack many proteins of lower mass, whereas in F9 there appeared to be an accumulation of very low mass proteins.

### Streptococcal proteins identified

In all, 19 different *S*. *pyogenes* proteins were identified in six of the fluid samples (Table 2 and Supplementary Table S1). All of the proteins were represented in the *emm*1 *S*. *pyogenes* database, however only streptococcal inhibitor of complement (SIC) and the hypothetical protein M5005_Spy0455 were unique to *emm*1. All other proteins were represented in most if not all other *emm*-type databases (Supplementary Table S1). Each identification appeared to be based on good quality data according to the associated metrics (Table 2 and Supplementary Table S1), nevertheless to further support the identifications, analysis of cognate bacterial supernatants was performed in parallel, to determine if the fluid proteins could be found in these samples as well. *S*. *pyogenes*-derived peptides identified in patient fluid samples corresponded to peptides from proteins also identified in cognate bacterial supernatants in all bar one case (Table 2 and Supplementary Table S2), thus these data further support the identification of 18 of the fluid proteins. Sixteen of the 19 *S*. *pyogenes* proteins were detected in fluid sample F8, two in fluid F5, and just one each in fluids F2, F4, F7 and F17.

**Table 2:** *S*. *pyogenes* proteins present in human fluid samples. The identification of each protein was determined by SEQUEST and was based on matching mass spectra of at least 2 different peptides for each protein. All proteins were matched to those from an *emm*1 strain, although (with the exception of inhibitor of complement protein and hypothetical protein M5005_Spy0455), others were also matched to most, and often all, other *emm* types examined, as detailed in Supplementary Table S1. The resultant probability calculation for each protein is given along with the number of peptides found. The peptide identifications were compared with results obtained from proteomic analysis of supernatant samples and in all cases, except for hypothetical protein M5005_Spy0455, between 1 and 9 peptides matching both supernatant and clinical fluid sample were found, providing additional confidence in these identifications.

Protein name	Locus tag	Fluid	Protein probability	No. of peptides	Common peptides
30S ribosomal protein S2	M5005_Spy1780	F8	2.5E-05	3	3/3
elongation factor G	M5005_Spy0232	F8	3.6E-06	2	2/2
elongation factor Ts	M5005_Spy1781	F8	4.9E-04	2	1/2
elongation factor Tu	M5005_Spy0508	F8	3.4E-12	9	9/10
exotoxin B/SpeB	M5005_Spy1735	F2	3.6E-08	3	3/3
fructose-bisphosphate aldolase	M5005_Spy1607	F8	4.9E-05	3	3/3
Gls24 family general stress protein	M5005_Spy0973	F8	1.5E-09	10	2/10
glyceraldehyde-3-phosphate dehydrogenase	M5005_Spy0233	F5	9.6E-14	2	2/2
glyceraldehyde-3-phosphate dehydrogenase	M5005_Spy0233	F8	1.4E-13	4	4/4
histidine triad protein	M5005_Spy1710	F8	1.6E-07	3	2/3
hypothetical protein	M5005_Spy0269	F8	1.2E-07	2	2/2
hypothetical protein	M5005_Spy0455	F4	3.1E-04	2	0/2
hypothetical protein	M5005_Spy0455	F7	6.1E-04	2	0/2
inhibitor of complement protein/SIC	M5005_Spy1718	F8	1.4E-09	3	3/3
manganese-binding protein	M5005_Spy0368	F8	4.0E-07	2	2/2
manganese-dependent inorganic pyrophosphatase	M5005_Spy0319	F8	8.1E-09	2	2/2
molecular chaperone GroEL	M5005_Spy1761	F8	2.2E-12	8	8/8
N-acetylmannosamine kinase	M5005_Spy0218	F17	6.0E-04	2	2/2
phosphoglycerate kinase	M5005_Spy1599	F8	2.0E-07	7	7/7
phosphopyruvate hydratase	M5005_Spy0556	F5	2.0E-08	4	4/4
phosphopyruvate hydratase	M5005_Spy0556	F8	1.1E-11	10	10/10
trigger factor	M5005_Spy1612	F8	3.7E-06	2	1/2

Fluid F8 was from a case of empyema caused by *emm*1 *S*. *pyogenes* and contained the highest copy number of *S*. *pyogenes* genomes among all the fluids tested. Among the 16 *S*. *pyogenes* proteins found in fluid F8 were several metabolic proteins and a number of virulence factors including the *emm*1-specific secreted virulence factor streptococcal inhibitor of complement (SIC)^12^, histidine triad protein^13^, as well as trigger factor^14^, which is required for maturation of the cysteine protease, exotoxin B (SpeB). SpeB was found in just one fluid, F2, from a case of necrotising fasciitis. Phosphopyruvate hydratase and glyceraldehyde-3-phosphate dehydrogenase were detected in both fluid samples F5 and F8. Interestingly, the hypothetical protein M5005_Spy0455, the gene for which is located adjacent to a toxin-antitoxin locus^15^, was detected in two fluid samples, F4 and F7, but was not identified in either of the corresponding bacterial supernatants, suggesting that expression of this protein may be upregulated *in vivo* compared with broth culture.

**Figure 2:**
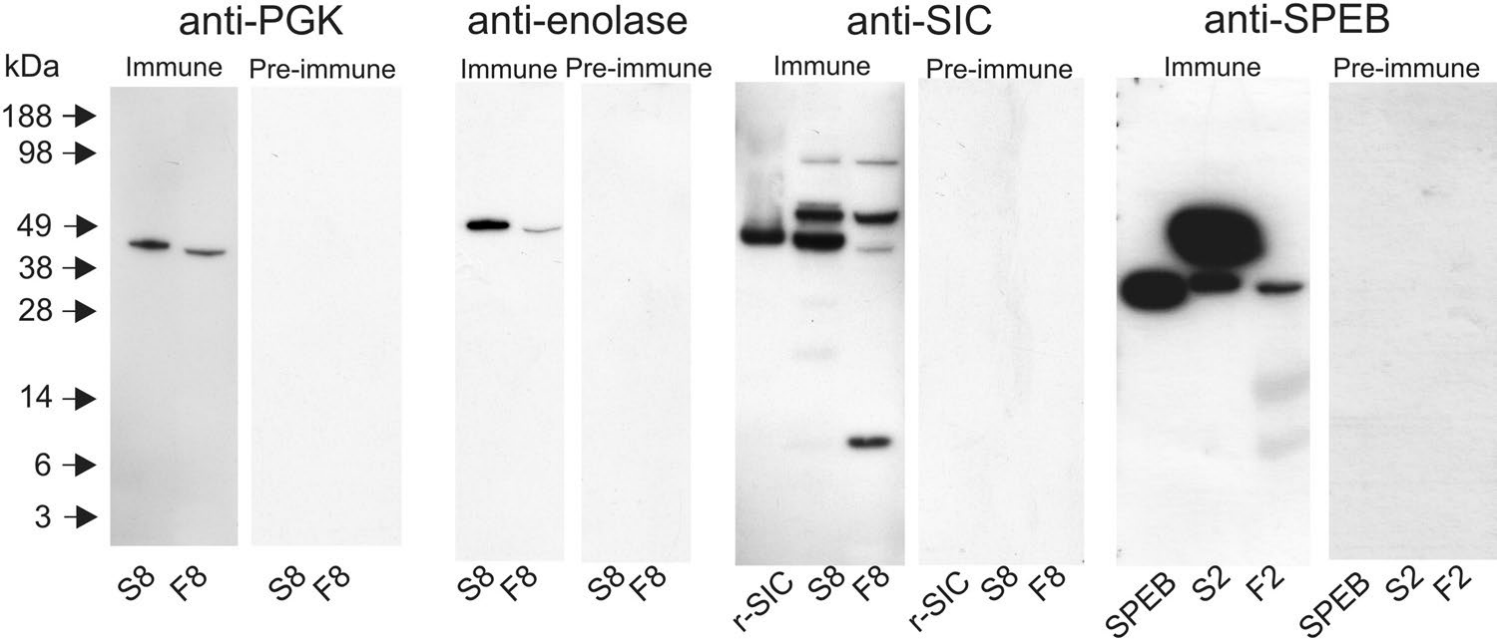
Immunoblotting of clinical fluid samples with CTAbs. The expression of PGK, enolase, SIC, and SpeB in fluid samples in clinical fluid samples was confirmed using antibodies against each of these antigens. Clinical fluid samples F2 and F8 along with corresponding cultured supernatant samples, S2 and S8, were prepared and analysed as described in the Methods section along with purified preparation of SIC and SpeB. The blots were developed with both a CTAb antiserum and corresponding pre-immune serum in each case to ensure that overdevelopment was avoided and to provide evidence that the observed immunoreactivity was antigen-specific. Blots developed using different antibodies are shown as clearly separate panels surrounded by white space; full length images of relevant lanes are shown.

To confirm the proteomic identification of a subset of proteins for which antibodies were available, immunoblotting was performed with C-terminal antibodies (CTAbs)^8^ against the *S*. *pyogenes* proteins phosphoglycerate kinase (PGK), phosphopyruvate hydratase (enolase), SIC, SpeB, glyceraldehyde-3-phosphate dehydrogenase, molecular chaperone GroEL, and elongation factor G. No immunoreactive bands were detected in any fluid sample using the antibodies against molecular chaperone GroEL or elongation factor G. The anti-PGK antibody recognised single bands in fluid sample F8 and the supernatant from the corresponding cultured streptococcal isolate (S8), and similarly the anti-enolase antibody revealed single bands in the same samples. The anti-SIC antibody recognised a single band corresponding to recombinant SIC and multiple bands in both fluid sample F8 and the supernatant S8 with a similar distribution, except that the fluid contained more of the band with the lowest mass. The anti-SpeB antibody recognised a single band corresponding to recombinant SpeB, and bands of equivalent mass in both fluid sample F2 and the corresponding supernatant S2, as well as to a band of slightly higher mass in the supernatant (Fig. 2). As such, the presence of PGK, enolase, SIC and SpeB in the fluid samples was supported by immunoblotting.

### Human proteins identified

**Figure 3:**
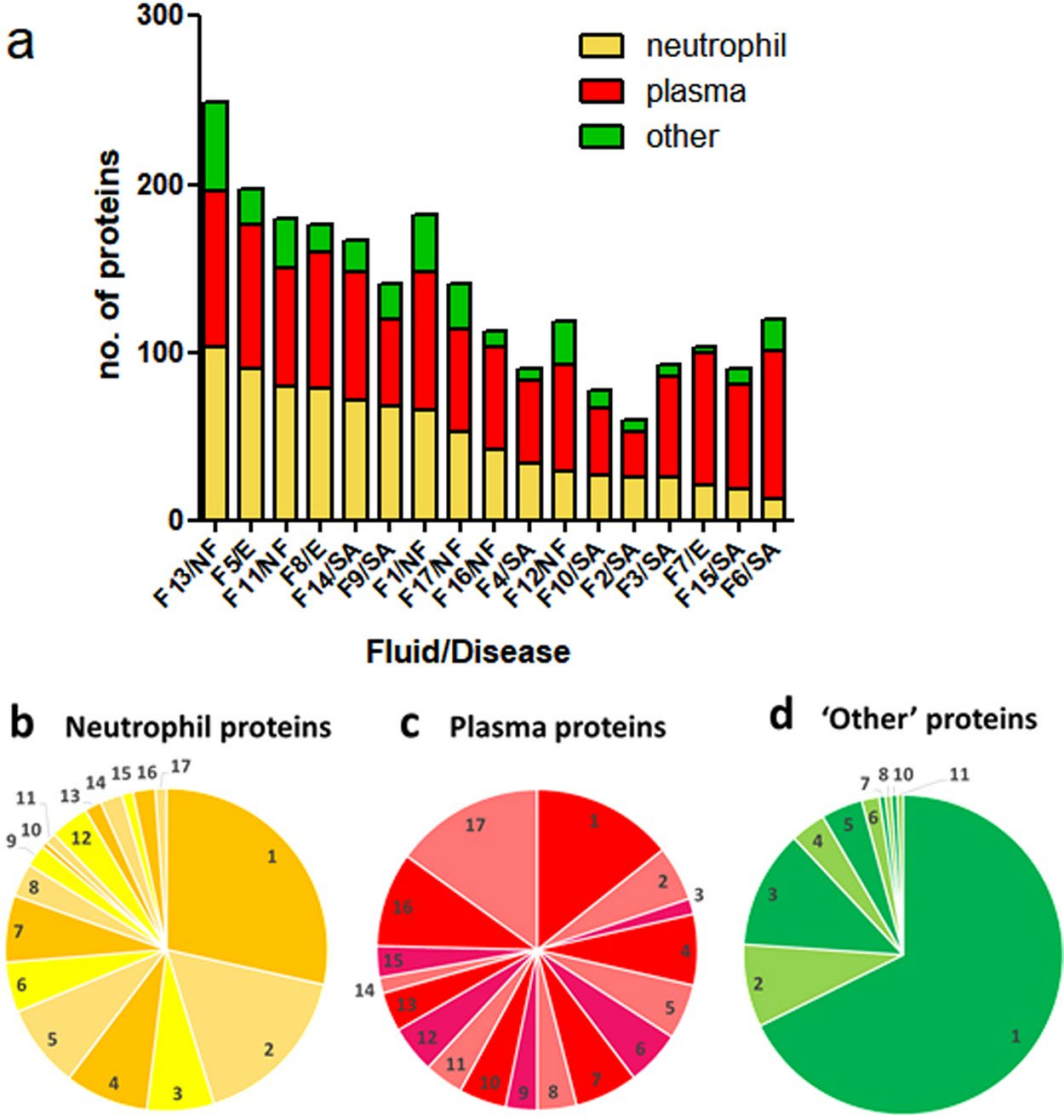
Comparative composition of human proteins in clinical fluid samples. Human proteins in each of the fluid samples were categorised as originating from neutrophils, plasma or neither of these sources (other). Categories were based on previous proteomic identifications and do not indicate that such proteins are limited to these sources. (**a**) The total number of proteins and the contribution from each of these groups is indicated. The order of the fluids have been arranged to highlight the variation in the number neutrophil proteins detected. (**b**–**d**) Pie charts showing the relative number of commonly found proteins in each category. Each figure represents groups of the number of proteins found to be common amongst the 17 fluids, with values ranging from 1 (proteins found in just one fluid sample) to 17 (proteins found in all 17 fluid samples).

**Figure 4:**
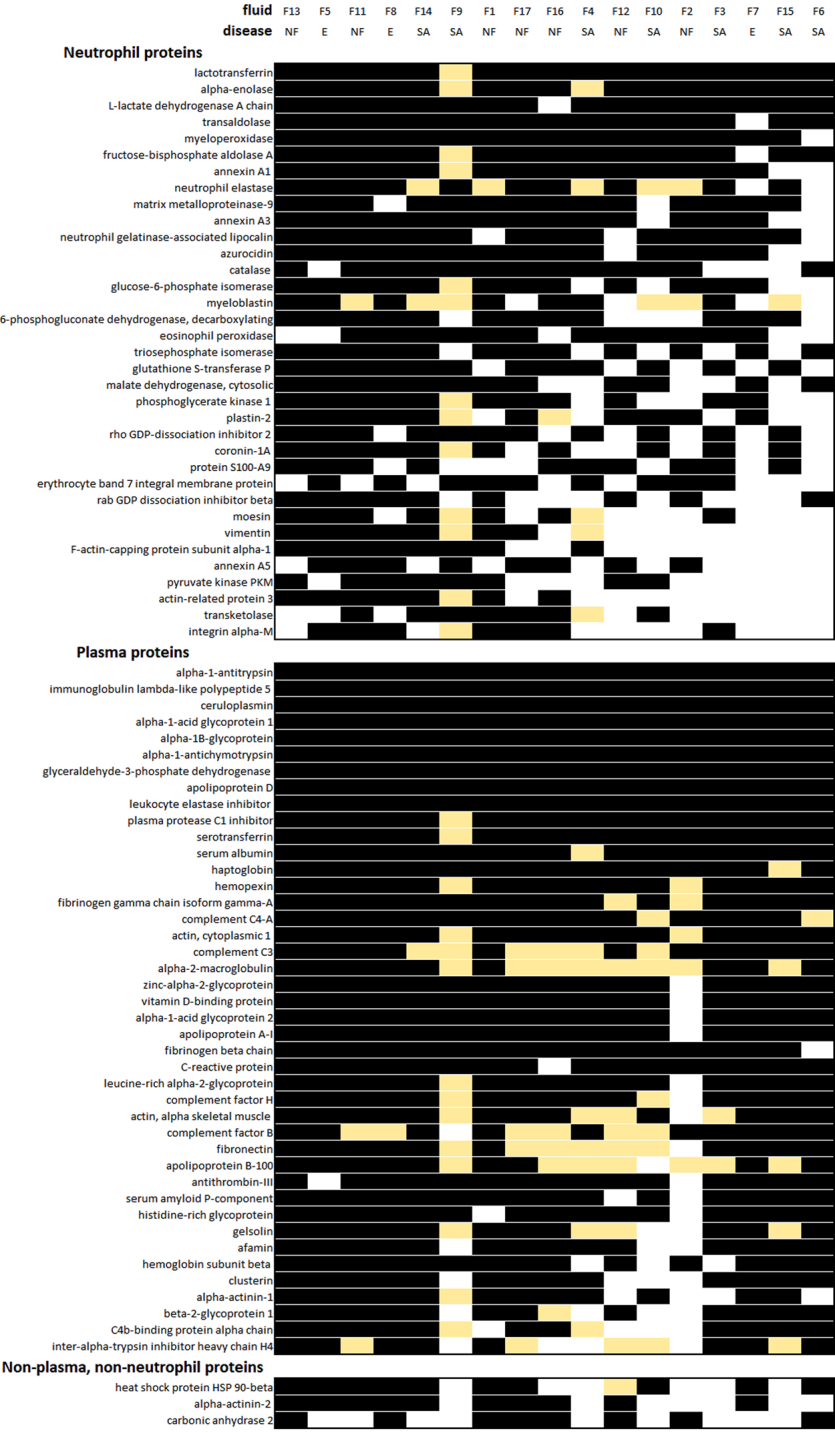
Distributions of the most commonly found human proteins in clinical fluid samples. Each coloured cell represents the presence of the protein indicated in clinical fluid samples F1–F17 (ordered so that those fluids with the greatest number of neutrophil proteins are to the left). The nature of the infection is indicated as necrotising fasciitis (NF), septic arthritis (SA), or empyema (E). Proteins identified in the clinical fluid samples were classified as either from neutrophil, plasma, or neither of these groups. Note that classifications do not indicate that such proteins are limited to these sources. Some proteins appeared with a lower mass than expected and these are indicated (yellow). Within each group the proteins are ordered with those proteins represented most frequently in the fluid samples placed at the top.

In total, 469 different human proteins were identified in the patient samples (Supplementary Table S3, Supplementary Table S4). Attempts to classify these proteins automatically based on processes available through PANTHER (http://www.pantherdb.org/) and DAVID (https://david-d.ncifcrf.gov/) proved unsuccessful, possibly due to the diversity of categories utilised and the lack of relevance to the samples being evaluated. However, an examination of proteins individually using resources such as WikiGenes (https://www.wikigenes.org) and UniProt (http://www.uniprot.org/) suggested that the proteins included many that originate from plasma as well as those involved in inflammation, particularly proteins produced by neutrophils. Consequently, we devised a more hypothesis-based approach where we tested this observation. This was achieved by comparing the fluid proteins identified with proteins previously identified in normal plasma that had been processed in the same way as the patient fluid samples (see Methods), and also by comparison with databases of proteins previously identified in neutrophils^16^. This approach appeared to be successful as it encompassed the majority of the proteins found (Fig. 3). Although numerically, fewer plasma proteins were detected compared with neutrophil proteins, the plasma proteins were more consistently represented in the fluid samples (Supplementary Table S5). Overall, there was considerable variability between the samples with regard to the number and categories of proteins identified (Fig. 3). Although the samples came from patients with empyema, arthritis, and necrotising fasciitis, there was no evident association between proteins identified in clinical samples and specific clinical disease phenotype. Notwithstanding the non-purulent appearance and serous nature of the clinical fluids, a large number of proteins previously identified in neutrophils^16^ were identified in the patient samples, consistent with the infective nature of the fluids. Two neutrophil proteins (lactotransferrin and alpha-enolase) were present in all samples (Fig. 4). The neutrophil protein, myeloperoxidase, was present in all fluid samples except fluid F6, which came from a patient with suspected reactive arthritis, and did not contain streptococcal DNA (Fig. 4, Table 1).

A number of human proteins, of both plasma and neutrophil origin, were shown to be present in a degraded form, potentially reflecting proteolysis *in vivo*. Of particular note, multiple human proteins in fluid F9, from a case of septic arthritis of the hip, were shown to be degraded, having migrated in SDS-polyacrylamide gel electrophoresis to a position corresponding to a mass that was lower than predicted. Although evidence of proteolysis could not be demonstrated, fluid F2, which contained the streptococcal cysteine protease SpeB, contained remarkably few plasma proteins at all in contrast to all other samples; many of those plasma proteins that were present in fluid F2 showed evidence of proteolysis, although we cannot exclude that this resulted from the actions of human neutrophil derived proteases (Fig. 4, Supplementary Table S3).

*S*. *pyogenes* virulence factors are widely reported to interact with a range of host proteins and, in some cases, can result in specific proteolytic cleavage. Plasma proteins that were identified at the sites of infection included fibronectin, all isoforms of fibrinogen, and complement factor H, although notably some of these were absent in some samples. Collagen I and IV were present in a minority of samples.

The properties of human proteins found in the fluid samples were examined by correspondence analysis, confirming the observed heterogeneity of samples with regard to disease phenotype and protein content (Supplementary Figure S2).

## Discussion

In this small but systematic study of samples obtained from the site of *S*. *pyogenes* infection, we were able to positively identify 19 different *S*. *pyogenes* proteins in a small number of clinical samples. The majority of bacterial proteins were accounted for by just one clinical sample from a patient with empyema caused by *emm*1 *S*. *pyogenes*. Similar to findings reported from earlier laboratory studies, *S. pyogenes* metabolic proteins that are normally considered to be cytosolic were identified most frequently. However, virulence-associated proteins such as SIC, SpeB, and trigger factor were also identified. In contrast to bacterial proteins, a number of human proteins common to neutrophils were identified in the clinical samples. Proteins such as lactotransferrin and alpha-enolase were reproducibly identified in all the samples, while myeloperoxidase was not identified in the absence of detectable bacterial DNA. Importantly, some samples showed evidence of proteolysis of human proteins.

To our knowledge, proteomic analysis of samples from patients with invasive *S*. *pyogenes* infection has not been previously attempted. Indeed, there are very few studies that have sought to identify bacterial proteins in samples from sites of infection using mass spectrometry (MS); the identification of bacterial proteins from mammalian hosts is considered to be difficult even in experimental *in vivo* models^17^. Many investigators have however undertaken proteomic or metabolomic analysis of serum or urine to distinguish patients with different categories of infection with varying degrees of success^18–21^. MALDI-ToF analysis of the bacterial proteome has revolutionised bacteriological colony identification in the clinical laboratory^22^, and there has been increasing interest in applying this approach to analysis of clinical specimens. However the number and quantity of bacterial proteins in human samples in comparison with the overwhelming number of human proteins makes such an approach challenging. Our aim in this work was therefore to identify dominant bacterial proteins that might act as targets for immunodiagnostics, where it is possible to identify analytes at a much lower concentration than would be feasible by liquid chromatography (LC) MS alone.

Surprisingly we identified a significant number of bacterial proteins, but in a limited number of samples. Although the sample with greatest bacterial abundance yielded several streptococcal proteins, this was not true for other samples that demonstrated high bacterial loads. We speculate that proteolysis may have reduced our ability to detect some bacterial proteins, while the timing of sampling and antibiotic exposure may have influenced this in other samples, notwithstanding the likely variable regulation of virulence by the pathogen itself. The findings suggest that immunoproteomic diagnostics targeted against *S*. *pyogenes* proteins may not be broadly effective as an approach. In contrast, molecular testing of samples for streptococcal DNA appeared to provide a potentially useful, quantitative result, and should be subject to further evaluation. Commercial immunodiagnostic tests for *S*. *pyogenes* pharyngitis rely upon detection of the streptococcal group A carbohydrate in throat swab samples; the samples used in this study were not tested for the presence of group A carbohydrate and thus we are unable to assess if such an immunodiagnostic might be a useful diagnostic for samples from invasive disease.

Although infrequent, the streptococcal proteins identified in clinical fluids offer insight into the pathogenesis of iGAS. Intriguingly both SpeB and trigger factor, required for post-translational activation of SpeB, were detected in necrotising fasciitis and empyema samples respectively, despite experimental data that demonstrate downregulation of SpeB in invasive infection when *S*. *pyogenes* virulence regulator *covR/S* mutants are under selective pressure^23^. The findings suggest that, during clinical invasive infection, homeostatic gene regulation may not result in such polarised effects as are seen in experimental settings, albeit that we have measured proteins that may accumulate in effusions at different stages of infection. It was recently reported that *S*. *pyogenes* upregulates around 15 key virulence proteins *in vitro*, in response to acid stress, including SpeB, SIC, and histidine triad protein, which is required for phagocytosis resistance^13^. It is notable then, that these same proteins were among the streptococcal proteins identified in clinical samples, in addition to the many metabolic proteins identified *in vitro*. Among the bacterial proteins identified were a number of hypothetical proteins of unknown function; these included Spy0455, the gene for which lies adjacent to a toxin-antitoxin locus^15^, that was only detected in clinical samples in two patients, yet was not detected at all during broth culture, suggesting that this protein may have a particular as-yet unrecognised function *in vivo*.

Development of bacterial diagnostics, therapeutics, and vaccines is contingent on reliable information regarding bacterial gene expression during clinical infection. Taken together, the bacterial proteomic data in this study underline the paucity of clinically-relevant proteomic information available regarding virulent bacterial infections in humans and highlight a need for more clinical studies. Our proteomic study provides a useful and systematic list of human proteins that are present at the sites of human group A streptococcal infection that will inform future studies of streptococcal pathogenesis. It is however unclear whether the human proteins identified in this study could act as alternative diagnostic targets for streptococcal, or other bacterial, infection, as the study was not designed to assess this. There is an imperative to improve diagnostic testing for septic conditions, in part to improve antimicrobial stewardship in hospitals in a setting where increasing antimicrobial resistance poses a major threat to healthcare. However, the rapidly lethal nature of invasive group A streptococcal infection provides a compelling rationale for a bespoke diagnostic test to assist in confirmation of disease. Based on the findings herein, DNA-based detection methods to detect pathogens such as *S*. *pyogenes* may be more successful than immunodiagnostic detection of bacterial proteins. Nonetheless, the proteomic findings related to both bacterial and host proteins provide useful insight for streptococcal pathogenesis research. Furthermore, although the use of leukocyte-derived products such as lactate dehydrogenase (LDH) to discriminate between inflammatory and non-inflammatory fluids is well-established, the data herein suggest that additional leukocyte biomarkers such as lactotransferrin, alpha-enolase, and myeloperoxidase deserve evaluation as potential biomarkers of infection.

## Methods

### Sample collection

Fluid samples (pleural fluid, tissue fluid, or joint fluid) aspirated from patients with suspected infection admitted to hospital between January 2008- February 2012 were submitted to the diagnostic laboratory for microscopy and culture. iGAS was diagnosed by positive identification of *S*. *pyogenes* from a normally sterile site, that is, either a blood culture or body fluid sample. Upon diagnosis of iGAS, fluid samples that had been refrigerated at 4 °C for a maximum of 72 h were transferred from the diagnostic laboratory to the research laboratory and frozen at −80 °C until analysed. Samples collected after 2010 (F1–F5; F9, F11) were treated with protease inhibitor cocktail (Calbiochem) before freezing; all samples in the study period were used bar those with a volume <1 mL.

### Ethical approval

The collection and analysis of biomarkers in clinical samples from patients with suspected infection was approved by the West London NHS Research Ethics Committee 06/Q0406/20; methods were in accordance with the specified approved protocol.

### Bacterial isolates

*S*. *pyogenes* isolates (n = 17) cultured from each patient were saved as glycerol stocks and frozen at −80 °C until required. Isolates were *emm* genotyped as described previously (Table 1)^24^. For proteomic analysis of bacterial culture supernatants, 7 isolates representing each of four different *emm* genotypes were cultured in 10 ml Todd Hewitt broth at 37 °C in 5% CO_2_. 4 ml supernatant from overnight culture was filtered (0.2 µm Sartorius, Germany) and proteins precipitated using 10%w/v TCA in acetone, washed with acetone, and dried for 1 h. The precipitate was dissolved in 250 µl of rehydration buffer (62.5 µl of 4 × LDS, 25 µl 1 M DTT and 162.5 µl of H_2_O). Proteomics was conducted on bacterial supernatants parallel with clinical samples.

### Quantification of *S*. *pyogenes* genomes in clinical samples by qPCR

DNA was extracted from 50 µl sample by 10 min heating at 95 °C followed by genomic DNA extraction (QiaAMP DNA mini kit, Qiagen). Knee fluid (F6) from a bacteremic patient that had failed to yield *S*. *pyogenes* and was believed to have a reactive, not septic, arthritis was used as a comparator. The *S*. *pyogenes* housekeeping gene *proS* was amplified using primers *ProS F* 5′TGAGTTTATTATGAAAGACGGCTATAGTTTC and *ProS R* 5′-AATAGCTTCGTAAGCTTGACGATAATC to generate a 93 bp product. Genomic copies of *proS* in each sample were quantified by comparison with standard concentrations of a plasmid containing a single copy of *proS* using protocols described previously^25^.

### SDS-polyacrylamide gel electrophoresis, in-gel tryptic digestion and LC-MS

Aliquots of each clinical sample containing 12.5 µg protein, except for sample 2 (25 µg) and sample 17 (6.25 µg), or 10 µg bacterial supernatant preparation, were treated with NuPAGE LDS Sample Buffer (ThermoFisher Scientific) containing 40 mM dithiothreitol and heated at 90 °C for 5 min. Iodoacetamide (200 mM) was added and incubated for 20 min prior to loading onto a 10% polyacrylamide Tris-glycine gel. Protein mass was estimated by comparison with the migration of the PageRuler Prestained Protein Ladder (ThermoFisher Scientific). Separated proteins were stained with Instant Blue (Expedeon) and an image produced using a laser scanner. Each lane in the gel was cut into 22 rows. The proteins present in each gel slice were digested with trypsin, and the resultant peptides extracted and analysed by LC-MS/MS as described previously^26^. The identification of proteins present in each gel slice was determined from analysis of the LC-MS/MS data acquired using an LTQ Linear Ion Trap Thermo Finnigan mass spectrometer coupled to an Agilent 1200 HPLC system for reverse phase nano LC-MS/MS peptide analysis. Precipitated bacterial proteins and human plasma samples were processed in a similar way.

### Proteomic analysis

To identify bacterial and human proteins, data were processed using SEQUEST utilising a combined protein database comprising sequences derived from nine *S*. *pyogenes* genomes, i.e. M1 (MGAS5005), M3 (MGAS315), M4 (MGAS10750), M5 (Manfredo), M6(MGAS10394), M12(MGAS2096), M28 (MGAS6180), M49(NZ131), M89 (H293), as well as human (refseq release 77), and pig trypsin. In addition, a decoy database was produced comprising the same composition, except with all sequences reversed. SEQUEST results were filtered based on peptide cross correlation scores exceeding 1.5 (single-charged ions), 2.0 (double-charged ions) and 2.5 (triple-charged ions) and identification of at least two different peptides to a protein with a probability score <0.01. Analysis using the decoy database indicated a false positive rate of 1.2%. For human protein identification, proteins were classified as neutrophil, plasma, or non-neutrophil/non-plasma by comparison with a published dataset of neutrophil proteins^16^ and an existing dataset of proteins identified in human plasma processed as above (Supplementary Table S5).

### Immunoblotting

Samples were separated on 10% bis-tris gels (Novex) and transferred to Hybond LFP membranes (GE Healthcare). Membranes were blocked with 5% skimmed milk (Sigma-Aldrich) prior to the addition of CTAb antibody^8^. Bound antibodies were detected using a 1:80,000 dilution of HRP-conjugated goat anti-rabbit IgG (Sigma-Aldrich) and the ECL prime detection system (GE Healthcare). Where appropriate, TCA-precipitated bacterial supernatants, or recombinant proteins, rSIC^24^ and rSpeB (Toxin Technology), were used as positive controls.

### Correspondence analysis

The occurrence of each protein in all of the fluid samples was considered with respect to the deduced coverage (based on the component tryptic peptides detected) and the position of that protein in the SDS-polyacrylamide gel (i.e. row position, which corresponds to relative protein mass). These criteria were used to classify each protein in all fluid samples using the following categories: Typical (where the row position that contained the maximum coverage of the protein was within an error of + or − 2 rows as that found in the majority of fluid samples), ‘high mass’ (where the row containing the maximum coverage was higher than Typical), ‘low mass’ (where the row containing the maximum coverage was less than Typical), ‘irregular’ (where no consistent row position corresponding to maximum coverage could be calculated), ‘single’ (if the protein was only detected in a single fluid sample), ‘neutrophil’ (if present in a previous proteomic analysis of neutrophils^16^), and ‘plasma’ (if the protein was also detected in normal human plasma samples). This was achieved algorthimically using an Excel spreadsheet. The resultant data was then tabulated and analysed using Correspondence Analysis (STATISTICA, Stat Soft Inc).

### Data availability

Data generated or analysed during this study are included in this published article (and its Supplementary Information files) or are available from the corresponding author on reasonable request.

## Electronic supplementary material

Table S1

Table S2

Table S3

Table S4

Table S5

Supplementary figures 1 and 2
