# Supplementary material for: Proteomic analysis at the sites of clinical infection with invasive Streptococcus pyogenes
Source: Sci Rep. 2018 Apr 13;8:5950. doi: 10.1038/s41598-018-24216-2 (PMC5899161; doi:10.1038/s41598-018-24216-2)

Supplementary Figures

Manuscript

Proteomic analysis at the sites of clinical infection with invasive *Streptococcus pyogenes*

Authors

Robert J Edwards, Marta Pyzio, Magdalena Gierula, Claire E Turner, Vahitha B Abdul-Salam and Shiranee Sriskandan

Supplementary Figure S1. Complete uncropped image of the SDS-polyacrylamide gel used to separate proteins prior to processing for proteomics. The prefix F indicates each of the fluid samples 1-17, and the prefix S indicates bacterial supernatant samples with the number indicating the corresponding fluid from which the culture was obtained. A protein molecular weight ladder (MWL) was applied at intervals. Unlabelled lanes were left intentionally empty as spacers or contain samples of no relevance to the current investigation.

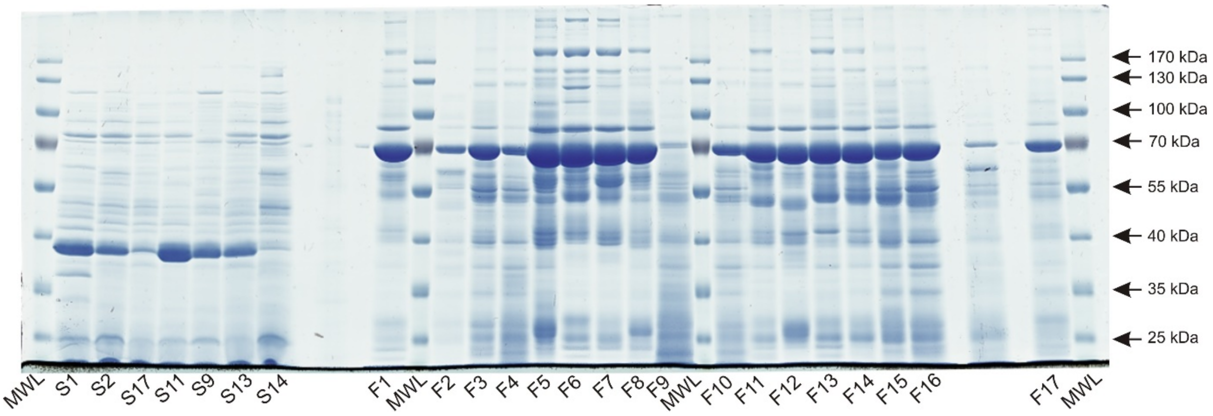

Supplementary Figure S2. Correspondence analysis of human proteins. Human proteins were categorised based on their known occurrence in neutrophils or plasma, and whether their mass and occurrence in each fluid sample corresponded to that of a typically found mass, a high mass, a low mass, an irregular pattern or only in a single fluid. These formed the categories for column data. The protein data in each fluid (or so-called rows) was comprised of 17 fluids which were from patients with either necrotising fasciitis, septic arthritis or empyema as indicated. Those fluids that contained proteins from *S. pyogenes* are indicted by asterisks. The main category of influence in dimension 1 is the low mass proteins and this appears to be a factor in F9. The categories of influence in dimension 2 in the positive direction are those proteins occurring in a single fluid and neutrophil proteins and F13 appears to share such characteristics. In the negative direction both the plasma protein and low mass categories are of importance which appears to explain the outlying data for fluids F3, F6, F7 and F15 and to a lesser extent fluids F4, F12 and F16.

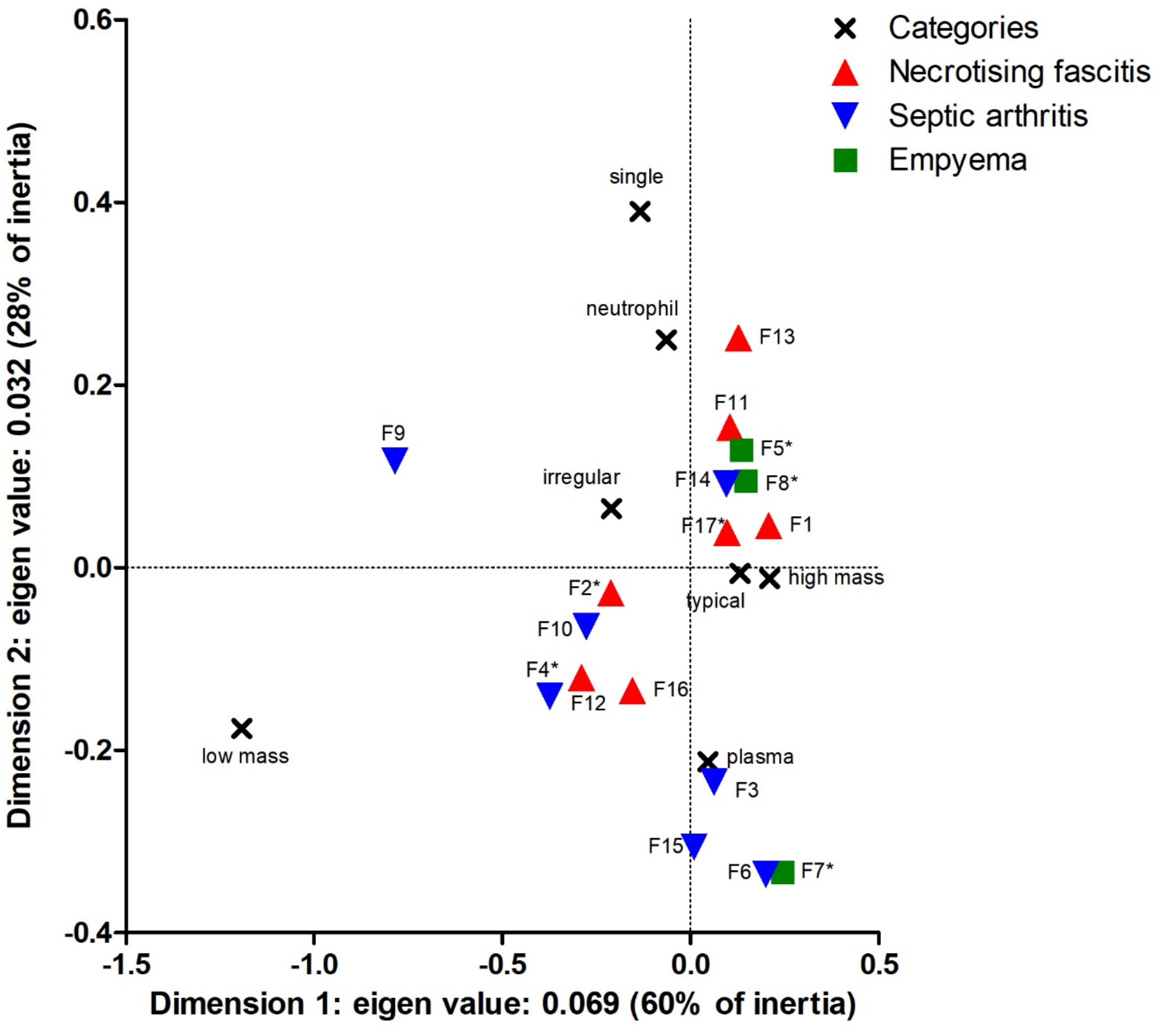

Supplement: Supplementary file 6 — Supplementary figures 1 and 2 [file 41598_2018_24216_MOESM6_ESM.pdf]
